# Carbidopa suppresses prostate cancer via aryl hydrocarbon receptor-mediated ubiquitination and degradation of androgen receptor

**DOI:** 10.1038/s41389-020-0236-x

**Published:** 2020-05-13

**Authors:** Zhiwei Chen, Aimin Cai, Hailun Zheng, Huirong Huang, Rui Sun, Xiao Cui, Weijian Ye, Qing Yao, Ruijie Chen, Longfa Kou

**Affiliations:** 1 Department of Pharmacy, The Second Affiliated Hospital and Yuying Children’s Hospital of Wenzhou Medical University, Wenzhou, Zhejiang 325027 China; 2 Wenzhou Municipal Key Laboratory of Paediatric Pharmacy, Wenzhou, 325027 China; 3 Department of Pharmaceutical Sciences, Wenzhou Medical University, Wenzhou, Zhejiang 325035 China

**Keywords:** Target validation, Apoptosis

## Abstract

Carbidopa, a peripheral decarboxylase inhibitor used with L-DOPA to treat Parkinson’s disease, has attracted significant interest in recent years for its anticancer effect. Increasing evidence reveals that Carbidopa can inhibit cancer cell growth and induce apoptosis through aryl hydrocarbon receptor (AHR) in some cancers. However, the antitumor effect of Carbidopa in prostate cancer (PCa) is not fully understood. Androgen receptor (AR) plays a central role in PCa, even in advanced “castrate-resistant” disease. In the present study, we report that Carbidopa suppresses the growth of PCa by downregulating the protein expression of AR. Carbidopa inhibits proliferation and migration of LNCaP cells and promotes apoptosis, but has no effect on the AR-independent prostate cell line DU145. Carbidopa increases ubiquitination of AR in LNCaP cells. Several studies have shown that AHR can act as an E3 ubiquitin ligase and promote the proteasomal degradation of AR. Quantitative RT-PCR, immunofluorescence staining and immunoblotting assay demonstrate that AHR is induced and activated by Carbidopa, and the co-immunoprecipitation assay shows that AR interacts with AHR, firmly confirming that Carbidopa decreases AR protein level though AHR-induced proteasomal degradation. In addition, Carbidopa suppresses PCa growth in vivo when xenografted into immunocompromised mice. Carbidopa treatment increases AHR protein level and decreases AR protein level in tumor tissues. Taken together, our study implicates Carbidopa for the first time in effective suppression of prostate cancer via a mechanism, involving AHR-mediated proteasomal degradation of AR.

## Introduction

Prostate cancer (PCa) is one of the most prevalent cancers in males^1^. Initially, the growth of prostate tumors is dependent on androgens that activate the androgen receptor (AR) in tumor cells, and promote their survival and proliferation^2^. Therefore, AR plays a key role in the development of PCa. Androgen deprivation therapy (ADT), the first-line treatment for metastatic PCa, primarily acts to reduce gonadal androgens through chemical or surgical castration, and/or disrupts AR signaling with the use of antiandrogens, such as bicalutamide^3^. Unfortunately, most PCas transform into castration-resistant PCa (CRPC) after ADT and ultimately lead to cancer recurrence, and AR is re-expressed and functions during this progression^4^. Despite the lack of dependence of CRPC on androgens, it is widely accepted that AR signaling still drives tumor growth^5^. Therefore, downregulation of AR can still be an effective means of treating PCa, even in castration resistance stage.

Carbidopa, a peripheral decarboxylase inhibitor, is used in combination with L-DOPA to treat Parkinson’s disease (PD)^6^. Recent studies have shown that Carbidopa has anticancer effects in many cancers, such as melanoma and pancreatic cancer^7,8^. Its anticancer effect may be related to the activation of AHR. AHR plays an essential role in maintaining cellular homeostasis^9^. It is overexpressed in multiple tumors, including pancreatic cancer, and hence, it is possible that AHR could prove to be an important drug target in some of these tumor types^10^. Carbidopa activates AHR by serving as its agonist ligand, thereby promoting its downstream target gene activation and inhibiting pancreatic cancer growth^7^. Thomas et al.^11^ showed that Carbidopa could enhance the antitumor effect of bicalutamide in CRPC. However, the molecular mechanisms responsible for the anticancer effects of Carbidopa in PCa remain largely unexplored, except for the finding that AHR is involved in the process.

There are several reports describing how AR is degraded and what chemical modifications of the AR protein mediate this phenomenon. AR can be phosphorylated by PI3K/AKT, then polyubiquitinated by MDM2 E3 ligase, and degraded through the 26 S proteasome proteolytic pathway^12^. Importantly, Ohtake et al.^13^ have reported that the ligand-activated AHR can promote the proteasomal degradation of sex steroid receptors, including ER and AR, and Sun et al.^14^ also found that icaritin could promote the AR degradation by activating AHR. Therefore, we hypothesized that the AHR-mediated proteasomal degradation of AR might be involved in Carbidopa-mediated suppression of the growth and proliferation of AR-positive PCa cells.

In this study, we show for the first time that Carbidopa, an activating ligand for AHR, inhibits PCa though AHR-mediated proteasomal degradation of AR. Carbidopa inhibits the growth and promotes apoptosis in PCa cells. These new data provide new insight into the anticancer efficacy of Carbidopa in PCa.

## Results

### Carbidopa inhibits LNCaP and VCAP cell proliferation and migration, and promotes apoptosis

**Fig. 1:**
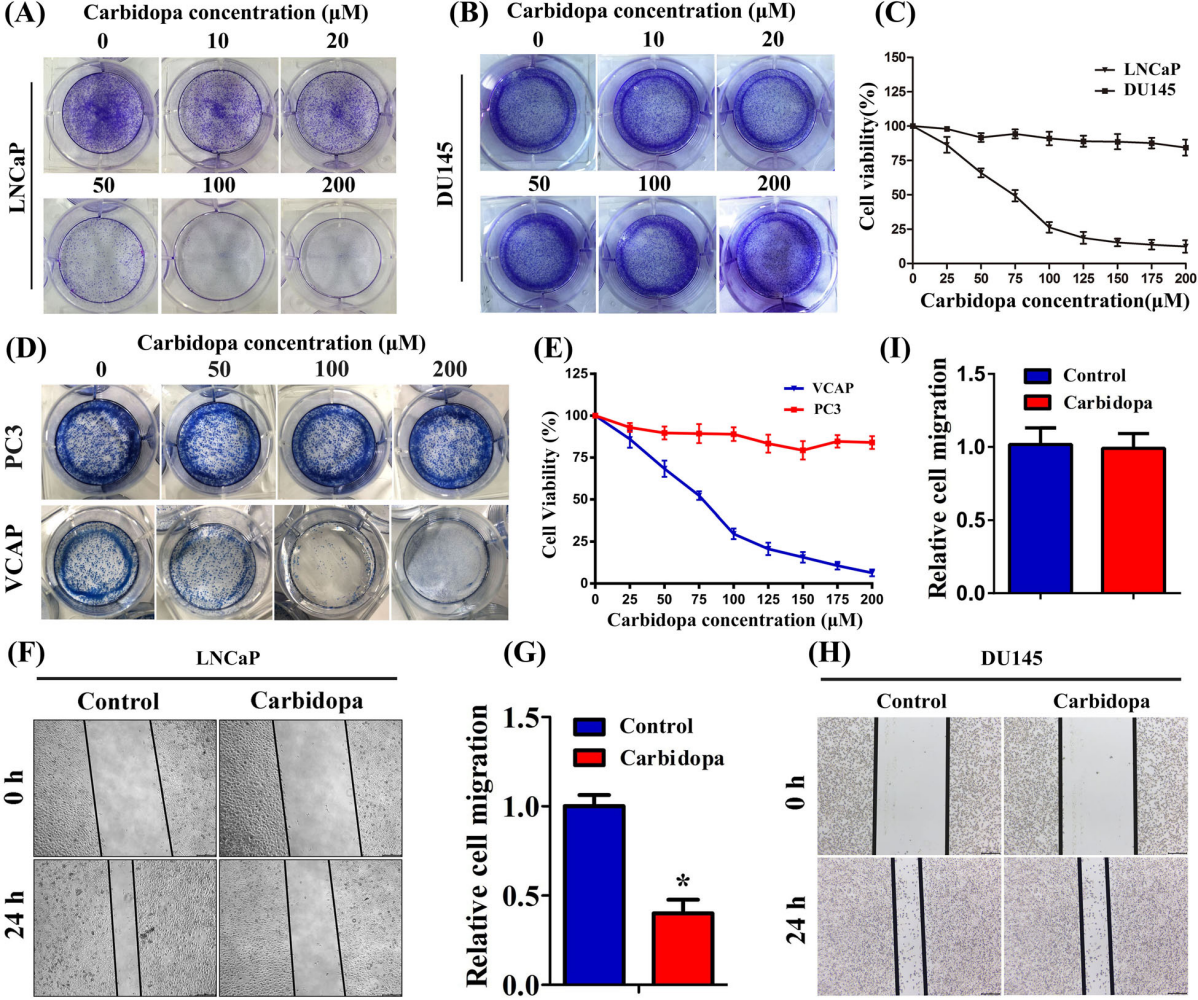
Carbidopa inhibits LNCaP cell proliferation and migration. **a**, **b** Colony formation assay in human PCa cell lines: LNCaP (**a**) and DU145 (**b**) cells were cultured in the presence of increasing doses of Carbidopa for 2 weeks and stained 0.1% crystal violet. **c** LNCaP and DU145 cells were treated with increasing doses of Carbidopa for 24 h, and the cell viability was examined by MTT assay. **d** Colony formation assay in human PCa cell lines: VCAP and PC3 cells were cultured in the presence of increasing doses of Carbidopa for 2 weeks and stained 0.1% crystal violet. **e** VCAP and PC3 cells were treated with increasing doses of Carbidopa for 24 h, and the cell viability was examined by MTT assay. **f** Migration assay analysis LNCaP cells with or without Carbidopa. **g** Quantitative analysis of cell migration in at least three separate fields; data are given as means ± SEM. *p < 0.05 versus control. **h** Migration assay analysis DU145 cells with or without Carbidopa. **i** Quantitative analysis of cell migration in at least three separate fields; data are given as means ± SEM.

**Fig. 2:**
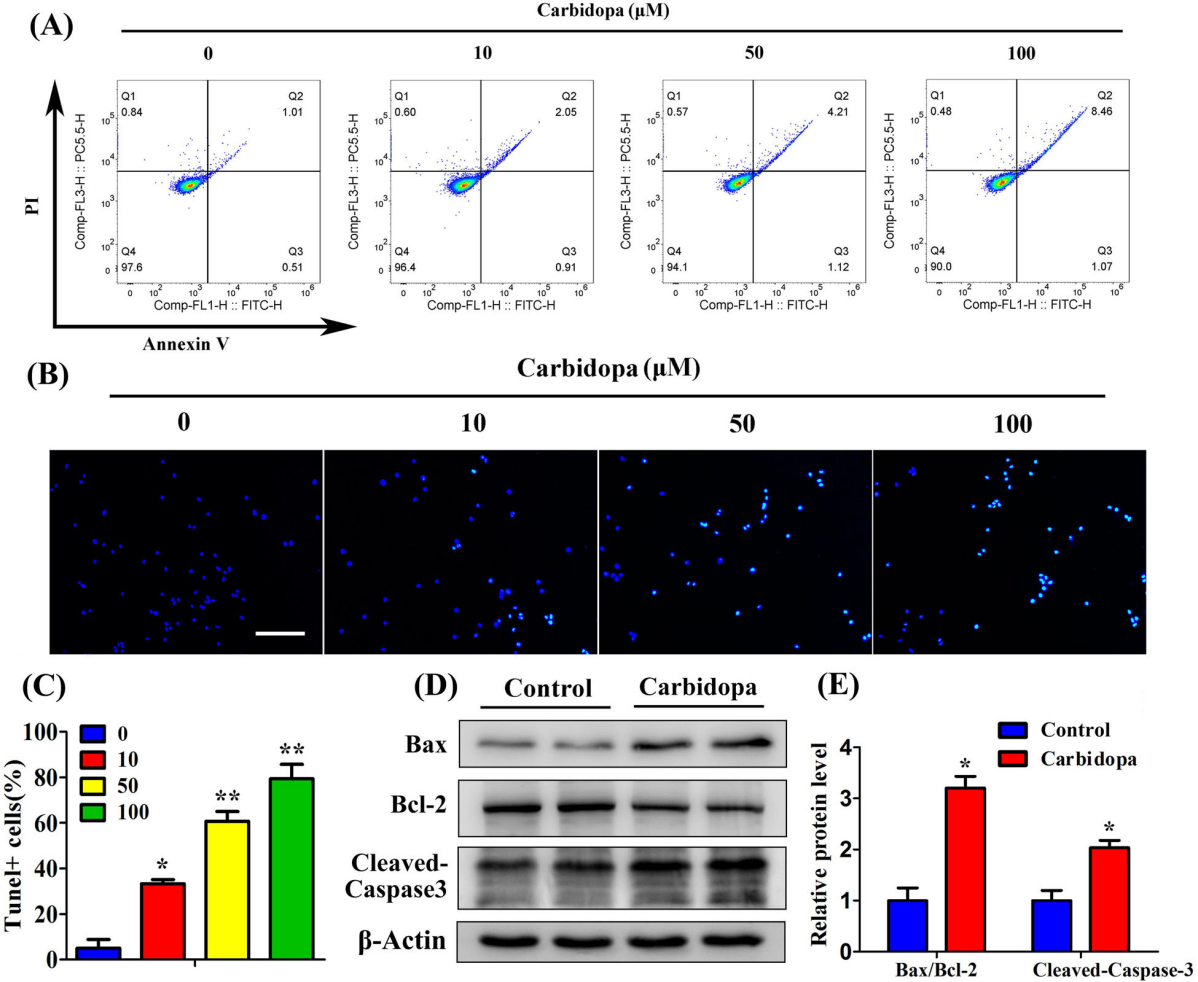
Carbidopa induces apoptosis in LNCaP cells. **a** Flow cytometry analysis of apoptosis in LNCaP cells with increasing doses of Carbidopa. **b** TUNEL assay in LNCaP cells. Cells were treated with increasing doses of Carbidopa; apoptotic cells were labeled green, and nuclei blue (DAPI). Figure shows merged images. Scale bars = 100 μm. **c** Quantitative analysis of TUNEL+ cells in at least six separate fields; data are given as means ± SEM. *p < 0.05 versus control. **p < 0.01 versus control. **d** Cell lysates of LNCaP cells were used to detect the Bax, Bcl-2, and cleaved caspase-3 protein levels by immunoblotting. **e** Quantitative analysis of each immunoblot; results were normalized to control and data are given as means ± SEM. *p < 0.05 versus control.

In order to determine whether Carbidopa can inhibit PCa growth, we used four human PCa cell lines in our study: LNCaP, VCAP, PC3, and DU145. LNCaP and VCAP cells are AR-dependent, whereas PC3 and DU145 cells are AR independent. The colony formation assay showed that Carbidopa significantly reduced the number of colonies in LNCaP and VCAP cells in a dose-dependent manner, while the colony numbers of DU145 cells and PC3 cells remained unaffected (Fig. 1a, b, d). We then used 3-(4,5-dimethylthiazol-2-yl)-2,5-diphenyltetrazolium bromide (MTT) assay to further validate the effect of Carbidopa on the proliferation of these four cell lines. As shown in Fig. 1c, e, Carbidopa inhibited the viability of LNCaP and VCAP cells in a dose-dependent manner, and the IC_50_ was 68 μM and 71 μM, respectively, whereas almost no effect on DU145 and PC3 was observed. We performed the cell migration assay to determine the effect of Carbidopa on the migrative ability of LNCaP cells. The cell migration of LNCaP and VCAP was significantly inhibited by the treatment of Carbidopa; the inhibition was >60% in the presence of 100 µM Carbidopa (Fig. 1f, g, Supplementary Fig. 1a, c). To determine the effect of Carbidopa on cell apoptosis, LNCaP, VCAP, PC3, and DU145 cells were treated with increasing doses of Carbidopa (10, 50, and 100 µM) for 24 h, and then cells were analyzed by flow cytometry. We found that apoptosis of LNCaP and VCAP cells was significantly induced by the treatment of Carbidopa (Fig. 2a, Supplementary Fig. 2b). However, there was no effect on PC3 and DU145 (Supplementary Fig. 2a, c). A tunel assay further confirmed the increased apoptosis in LNCaP cells (Fig. 2b, c). Moreover, Carbidopa elevated protein levels of cleaved Caspase-3 (c-Caspase-3) and Bax/Bcl-2 in LNCaP cells (Fig. 2d, e). Taken together, the results indicate that Carbidopa functions as a negative growth regulator for the LNCaP and VCAP cells, but not for the DU145 and PC3 cells.

### Carbidopa decreases the AR protein levels through the ubiquitin-proteasome pathway

**Fig. 3:**
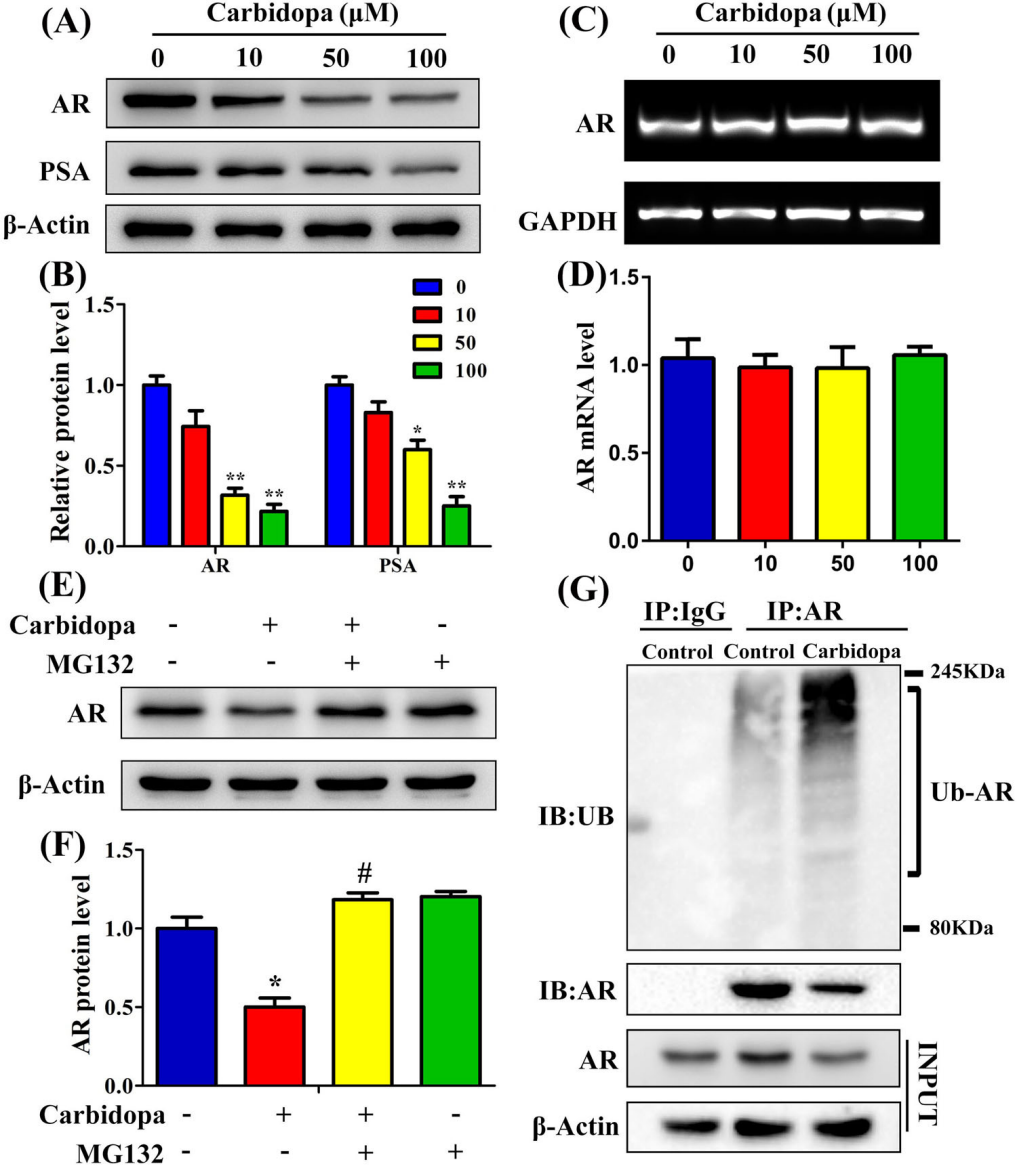
Carbidopa decreases AR protein levels through ubiquitin-proteasome pathway. **a** Immunoblot analysis of AR and PSA. LNCaP cells were treated with increasing doses of Carbidopa for 24 h. **b** Quantitative analysis of each immunoblot; results were normalized to control and data are given as means ± SEM. *p < 0.05 versus control. **p < 0.01 versus control. **c** RT-PCR analysis of AR mRNA. **d** Quantitative RT-PCR analysis of AR mRNA level. LNCaP cells were treated with Carbidopa for 24 h. GAPDH was used as the loading control. Results were normalized to control and data are given as means ± SEM. **e** Immunoblot analysis of AR. LNCaP cells were incubated in the presence or absence of Carbidopa (100 μM) for 24 h, and in the final 6 h of treatment, the cells were treated with or without 10 μM MG132. **f** Quantitative analysis of each immunoblot; results were normalized to control and data are given as means ± SEM of three independent experiments. *p < 0.05 versus control. ^#^*p* < 0.05 versus Carbidopa treatment. **g** LNCaP cells were incubated in the presence or absence of Carbidopa (100 μM) for 24 h. AR was immunoprecipitated, and the immunoprecipitated was then used for immunoblot to analyze ubiquitinated AR.

AR plays a central role in the proliferation of PCa cells and regulates various androgen target genes, such as prostate-specific antigen (PSA)^15^. As the inhibitory effects of Carbidopa were noticed only in AR-dependent LNCaP and VCAP cells, and not in AR-independent DU145 and PC3 cells, we suspected that Carbidopa might have a selective impact on AR in PCa cells. To test this idea, we first evaluated the AR protein levels in four prostate cell lines; we found the receptor protein only in LNCaP and VCAP cells (Supplementary Fig. 3). PC3 and DU145 cells did not have the AR protein. We then focused on cellular levels of AR protein in LNCaP and VCAP cells, with and without Carbidopa treatment. We found that Carbidopa decreased the expression of AR in LNCaP and VCAP cells (Fig. 3a, b, Supplementary Fig. 4a, b); in parallel, the expression of PSA, a known target gene for AR, was also decreased in LNCaP cells (Fig. 3a, b). To our surprise, there was no significant change in mRNA levels of AR in response to Carbidopa treatment (Fig. 3c, d). Therefore, we speculated that Carbidopa might directly inhibit the level of AR protein by means of protein degradation, with no effect at the transcription level. The ubiquitin-proteasomal pathway has been shown to mediate AR degradation, involving Mdm2, an E3 ubiquitin ligase^12^. Thus, we hypothesized that Carbidopa decreased the AR protein levels through ubiquitin-proteasome pathway. To confirm this, LNCaP cells were incubated in the presence or the absence of Carbidopa for 24 h, and in the final 6 h of the treatment, the cells were treated with or without 10 μM MG132, a ubiquitin-proteasome inhibitor. As shown in Fig. 3e, f, treatment with MG132 prevented the Carbidopa-induced downregulation of AR protein. Then, we checked whether AR was ubiquitinated after Carbidopa treatment. As shown in Fig. 3g, Carbidopa treatment increased the level of ubiquitinated AR. These results suggested that Carbidopa decreased the levels of the AR protein and that the effect occurred mainly via the ubiquitin-proteasome pathway.

### Carbidopa is not just an activating agonist for AHR; it also induces the expression of AHR both at the mRNA level and the protein level

**Fig. 4:**
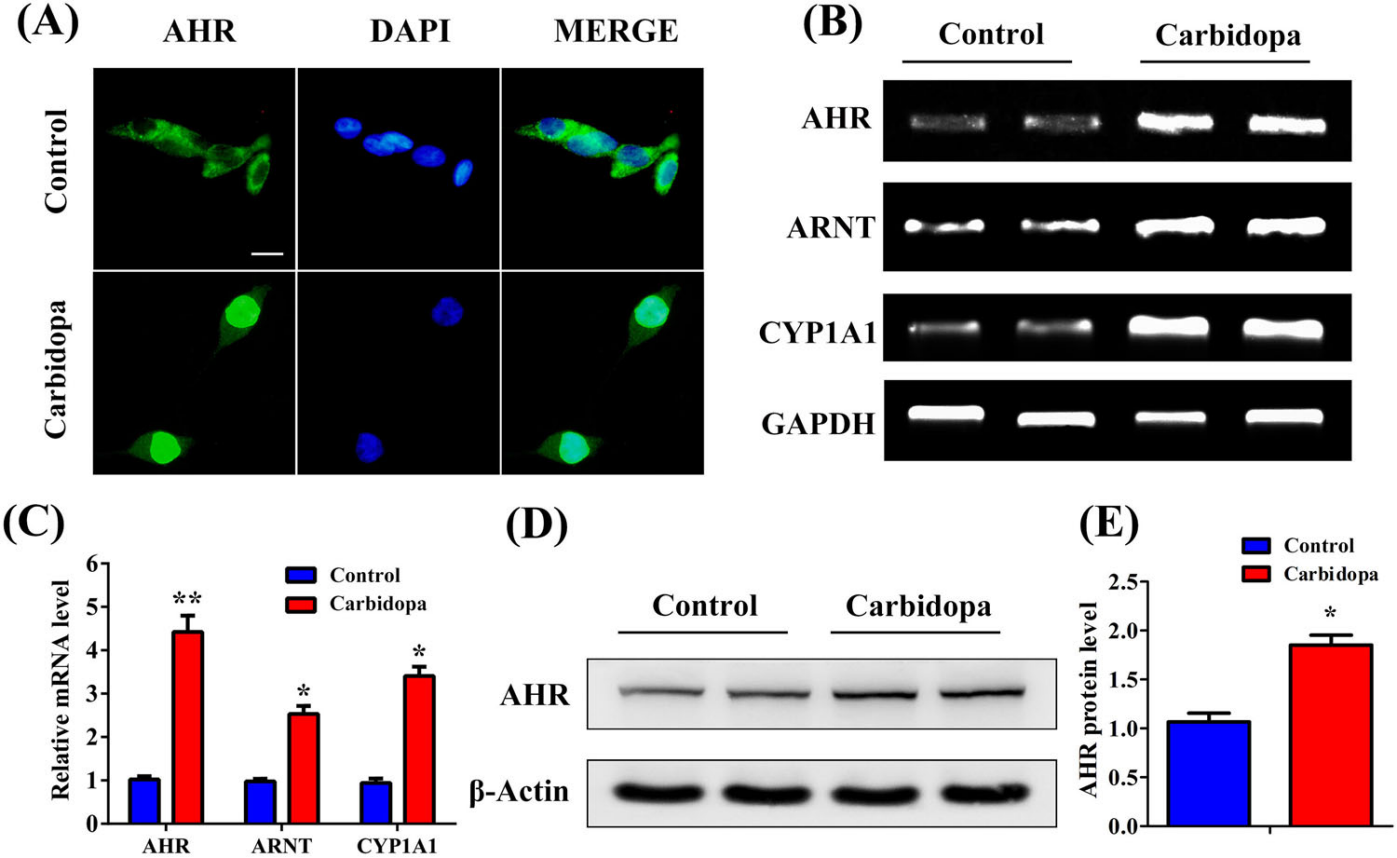
Carbidopa promotes translocation of AHR protein to nucleus and also increases AHR mRNA levels in LNCaP cells. **a** Immunofluorescence staining to determine subcellular localization of AR. LNCaP cells were treated with or without Carbidopa (100 μM) for 24 h. Scale bars = 50 μm. **b** RT-PCR analysis of AHR, ARNT, and CYP1A1 mRNA levels. LNCaP cells were treated with or without Carbidopa (100 μM) for 24 h. **c** qRT-PCR analysis of the mRNA level of AHR, ARNT, and CYP1A1. LNCaP cells were treated with or without Carbidopa. GAPDH was used as the loading control. Results were normalized to control and data are given as means ± SEM of three independent experiments. *p < 0.05 versus control. **p < 0.01 versus control. **d** Immunoblot analysis of AHR protein. LNCaP cells were treated with or without Carbidopa (100 μM) for 24 h. **e** Quantitative analysis of each immunoblot; results were normalized to control and data are given as means ± SEM of three independent experiments. *p < 0.05 versus control.

It has been reported that the ligand-activated AHR can promote the proteasomal degradation of sex steroid receptors, including ER and AR^13^. Therefore, we tested the effect of carbidopa on ER using MCF7 cells, and we found a similar suppressing effect on ER (Supplementary Fig. 5). Given that Carbidopa suppressed the expression of AR through ubiquitin-proteasomal pathway, we explored whether Carbidopa can also induce AHR in addition to its already documented role as an activating agonist ligand. We analyzed the mRNA level of AHR and its transcriptional target genes. As shown in Fig. 4a, b, the mRNA expression of AHR, ARNT, and CYP1A1 were significantly elevated after Carbidopa treatment. Unliganded AHR resides in the cytoplasm, forming a complex with heat shock proteins^16^. Upon binding an agonist, the cytoplasmic complex dissociates, and the ligand-bound AHR gets translocated to the nucleus. Ligand-dependent activation of AHR is associated with the translocation of the receptor from the cytoplasm into the nucleus^17^. Therefore, we used immunofluorescence to localize AHR in control and Carbidopa-treated LNCaP cells. The result suggested that Carbidopa treatment led to the nuclear translocation of AHR. Furthermore, we evaluated the protein level of AHR in LNCaP cells following the treatment with or without 100 μM Carbidopa. The immunoblotting study showed that Carbidopa increased the levels of AHR protein (Fig. 4d, e). Accordingly, Carbidopa not only activates AHR by serving as its ligand, but also induces the expression of AHR in LNCaP cells.

### Carbidopa inhibits PCa via AHR-mediated AR destabilization

**Fig. 5:**
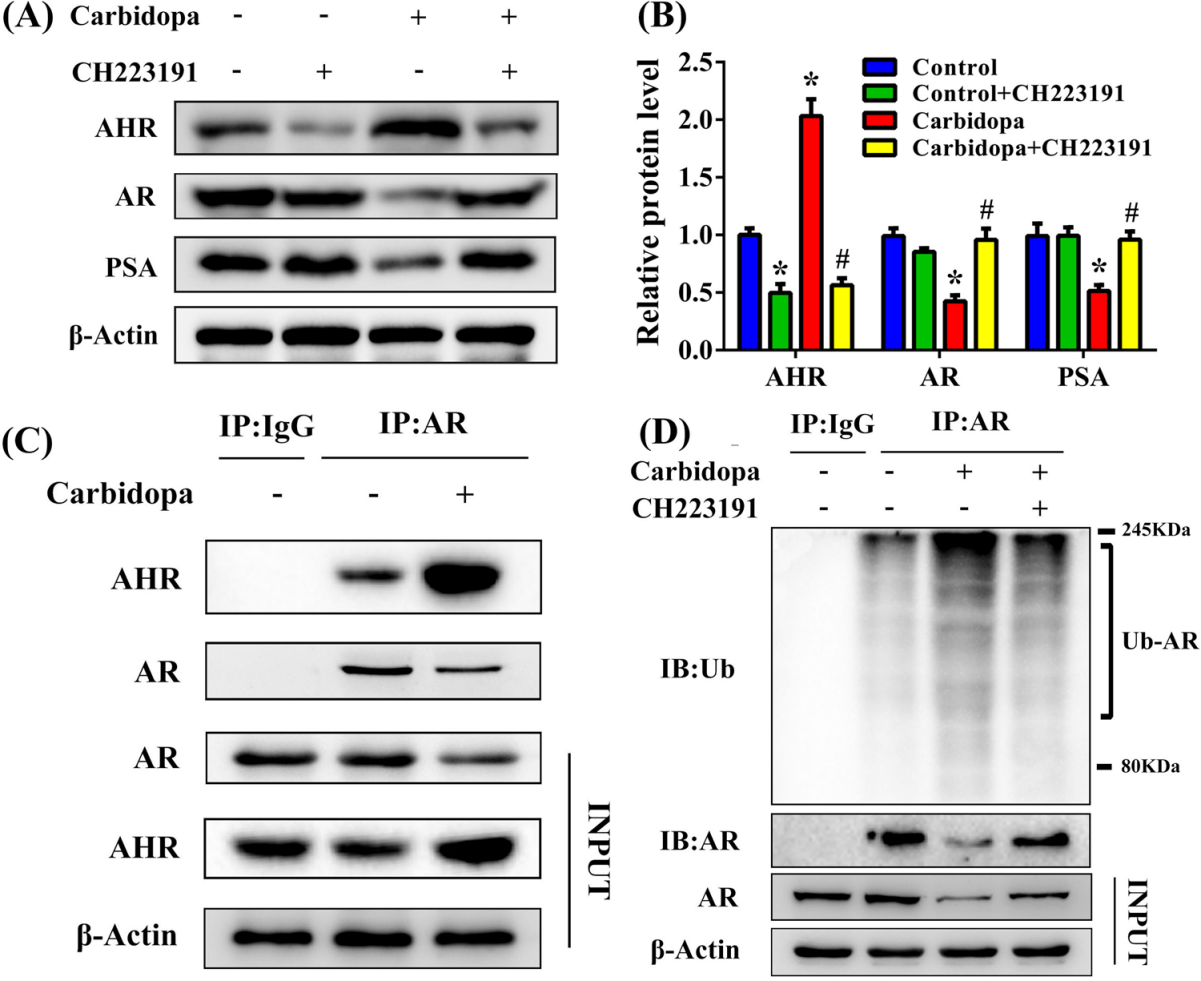
Carbidopa decreases AR protein levels via AHR-mediated proteasomal degradation. **a** Immunoblot analysis of AR, PSA, and AHR. LNCaP cells were treated with or without Carbidopa for 24 h. For blocking AHR, CH223191 (10 μM) was added in the experiment as indicated. **b** Quantitative analysis of each immunoblot; results were normalized to control and data are given as means ± SEM of three independent experiments. *p < 0.05 versus control. ^#^*p* < 0.05 versus Carbidopa treatment. **c** LNCaP cells were treated with or without Carbidopa (100 μM). Co-immunoprecipitation assay was used to analyze the interaction between AHR and AR. Immunoprecipitation was done with AR antibody and immunoblot was carried out for AR and AHR. **d** LNCaP cells were treated with or without Carbidopa (100 μM). CH223191 (10 μM) was co-treated with Carbidopa where indicated. Co-immunoprecipitation assay was used to analyze ubiquitinated AR. Immunoprecipitation was done with AR antibody and immunoblot was carried out for ubiquitin.

We then wanted to see whether Carbidopa inhibited PCa through AHR-mediated AR degradation. We monitored the involvement of AHR in the effects of Carbidopa by using an AHR-specific antagonist, CH223191. LNCaP cells were incubated with 100 μM Carbidopa and then treated with or without CH223191. The protein levels of AR and PSA were dramatically increased after the treatment with CH223191 compared to treatment with only Carbidopa (Fig. 5a, b). We preliminarily concluded that the downregulation of AR protein level in response to the Carbidopa treatment required AHR. It has been reported that the AHR can promote the proteasomal degradation of AR^18^. Therefore, we used the co-immunoprecipitation assay to demonstrate protein–protein interaction between AR and AHR. The results of these experiments indicated that AHR physically interacts with AR (Fig. 5c). We then checked Carbidopa-induced AR ubiquitination after CH223191 treatment. As shown in Fig. 5d, CH223191 downregulated Carbidopa-induced AR ubiquitination. These results suggested that Carbidopa decreased AR via AHR-induced proteasomal degradation.

**Fig. 6:**
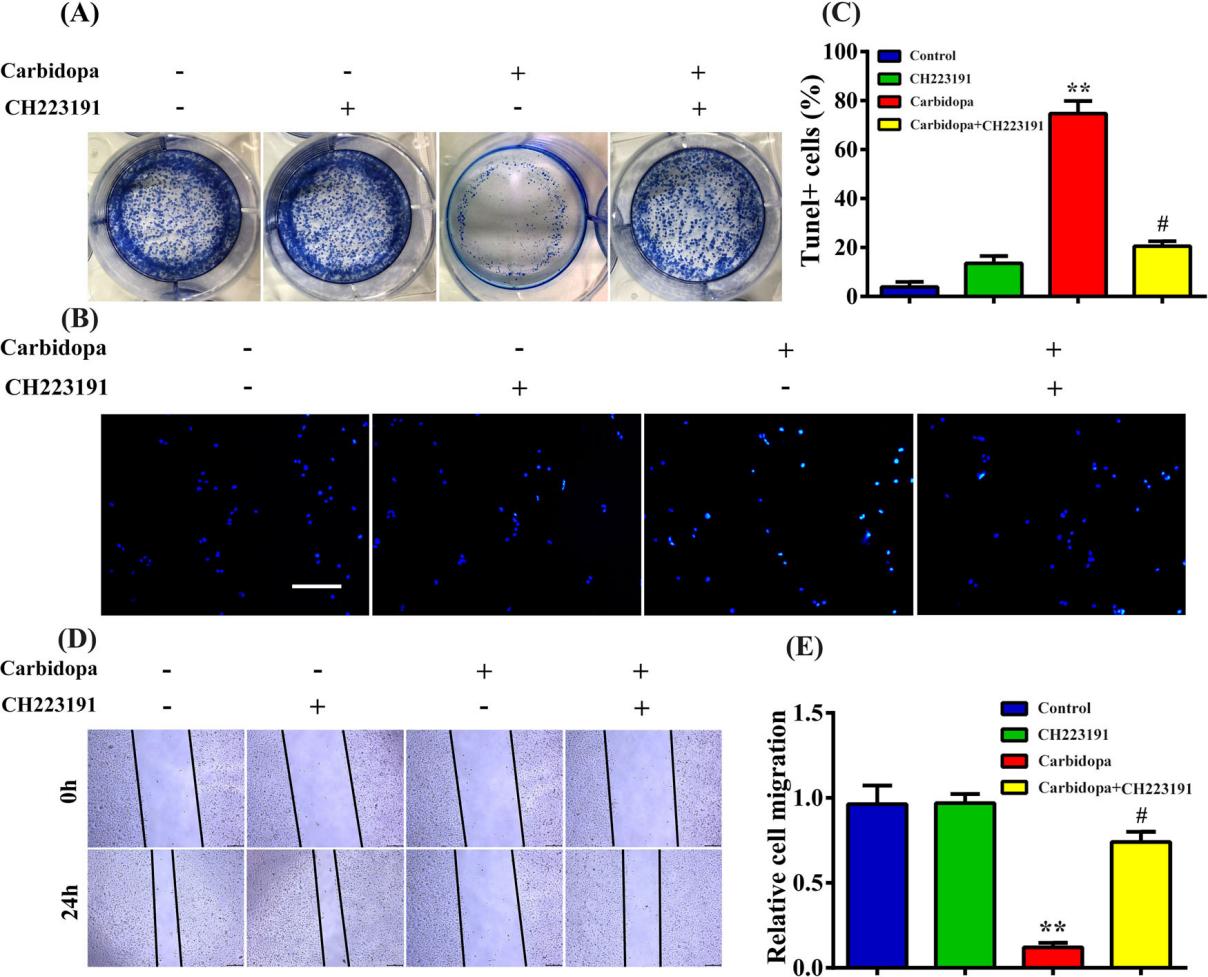
Carbidopa inhibits proliferation of LNCaP cells via AHR. **a** Colony formation assay: LNCaP cells were treated with or without Carbidopa (100 μM). Where indicated, AHR blocker CH223191 was present (10 μM). **b** TUNEL assay: LNCaP cells were treated with or without Carbidopa (100 μM). Where indicated, AHR blocker CH223191 was present (10 μM). Scale bars = 100 μm. **c** Quantitative analysis of TUNEL+ cells in at least three separate fields; data are given as means ± SEM. **p < 0.01 versus control. ^#^*p* < 0.05 versus Carbidopa treatment. **d** Migration assay: LNCaP cells were treated with or without Carbidopa (100 μM). Where indicated, AHR blocker CH223191 was present (10 μM). Scale bars = 100 μm. **e** Quantification of the migration assay. Data are given as means ± SEM of three independent experiments. **p < 0.01 versus control. ^#^*p* < 0.05 versus Carbidopa treatment.

We then wanted to explore whether Carbidopa inhibited the growth of PCa with an obligatory involvement of AHR. As shown in Fig. 6, Carbidopa-induced inhibition of cell proliferation and migration were reactivated by CH223191. Furthermore, CH223191 restored Carbidopa-induced apoptosis. Taken together, the data lead to the conclusion that Carbidopa inhibited PCa by AHR-mediated AR ubiquitination and consequent degradation.

### Carbidopa suppressed PCa growth in mice

**Fig. 7:**
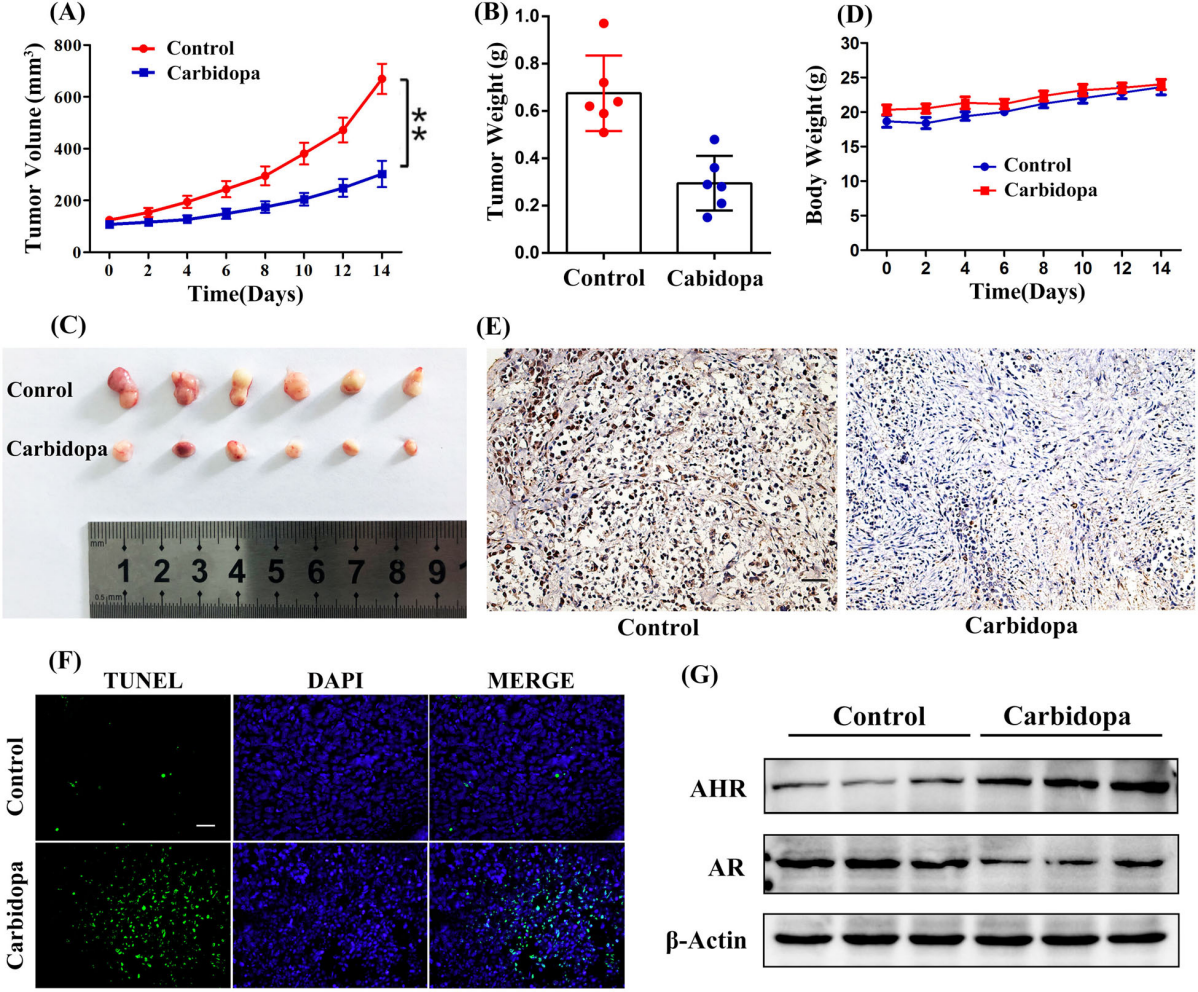
Carbidopa inhibits PCa in nude mice. **a** Measurement of tumor volumes at indicated time points, following implantation of LNCaP cells in BALB/c nude mice. The nude mice were treated with 7.5 mg/kg and equal volume of vehicle. Data are given as means ± SEM, *n* = 6. **p < 0.01 versus control. **b** Measurement of harvested tumor weights at the conclusion of the study. Data are given as means ± SEM, *n* = 6. **p < 0.01 versus control. **c** Photographs of resected tumor tissues from BALB/c nude mice at the conclusion of the study. **d** Measurement of body weights at the conclusion of the study. Data are given as means ± SEM, *n* = 6. **e** Immunohistochemically staining of tumor specimens for cell proliferation marker Ki67. Immunoreactivity was detected by DAB chromogen (brown). Scale bars = 100 μm. **f** TUNEL assay in tumor specimens. Apoptotic cells were labeled green, and nuclei blue (DAPI). Figure shows merged images. Scale bars = 100 μm. **g** Immunoblot analysis of AR, and AHR in tumor specimens.

Finally, we assessed the in vivo anti-PCa effect of Carbidopa using a human PCa xenograft mouse model. LNCaP cells were implanted in BALB/c nude mice, followed with intraperitoneal (i.p.) treatment of Carbidopa. It was evidenced that Carbidopa significantly suppressed the tumor growth compared to the control group (Fig. 7a–c). Negligible variation of the body weight was observed during the treatment (Fig. 7d). Immunohistochemical analysis was performed to detect the expression of Ki-67, a key marker for cellular proliferation. The results showed a profound decrease of Ki-67 expression after Carbidopa treatment (Fig. 7e). The proapoptotic effects of Carbidopa were demonstrated by tunel staining (Fig. 7f). We further evaluated the AHR and AR protein level in these tumor tissues using immunoblot assay (Fig. 7g). It was clear that Carbidopa increased the levels of AHR and decreased the levels of AR. These results were consistent with those in LNCaP cells, suggesting Carbidopa inhibited PCa via AHR-mediated ubiquitin-proteasomal degradation of AR.

## Discussion

**Fig. 8:**
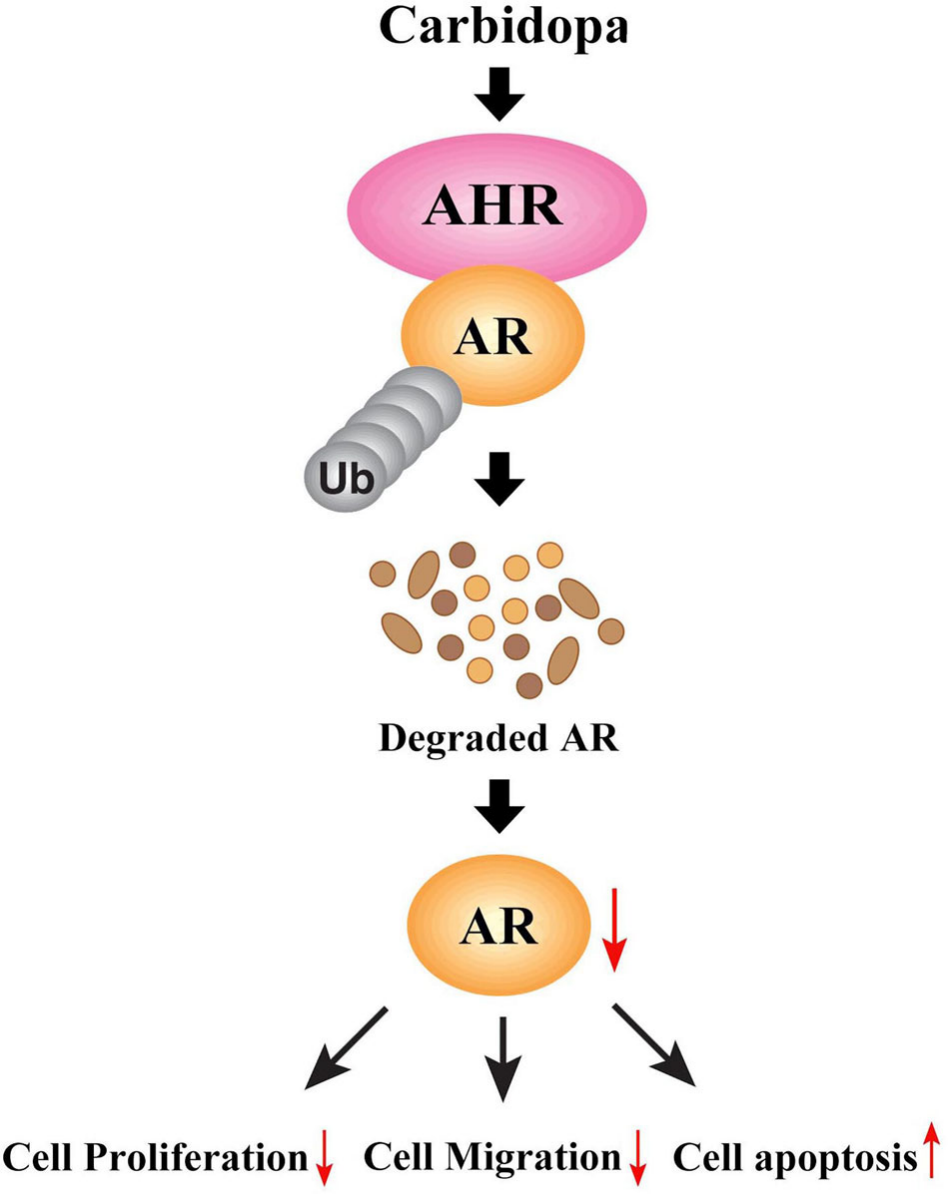
Schematic showing the proposed mechanism of Carbidopa in suppressing the growth and proliferation of AR-dependent LNCaP cells. Carbidopa could induce the activation of AHR and resultant ubiquitination and degradation of AR, therefore inhibiting proliferation and migration of AR-dependent prostate cancer cells and promoting apoptosis.

Several studies have revealed that Carbidopa could induce apoptosis in many types of cancer cells in vitro, and inhibit the growth of tumors with transplanted human cancer cell lines in vivo^7,19^, but the molecular mechanism of Carbidopa in anticancer processes, especially in anti-PCa process, is still not well known. This study provides novel evidence that Carbidopa exerts its potent antiproliferative and proapoptotic effects on PCa both in vitro and in vivo. The effect of Carbidopa on apoptosis of PCa may be due to the upregulation of apoptosis-related proteins expression (Fig. 2d). Carbidopa effectively suppressed AR protein level through acceleration of the AHR-mediated proteasomal degradation of proteins in LNCaP cells (Fig. 8). Carbidopa was shown to significantly reduce the growth rate of androgen-dependent LNCaP and VCAP cells (Fig. 1a, Supplementary Fig. 1). In contrast, Carbidopa did not affect the proliferation of AR-negative DU145 and PC3 PCa cells (Fig. 1b), suggesting that the effect of Carbidopa requires the presence of AR. More importantly, the mRNA level of AR has slightly changed when treated with Carbidopa (Fig. 3c). That means the inhibitory effect of Carbidopa on AR was not achieved at the transcriptional level. This could be a new, hitherto unknown, signaling pathway for the efficacy of Carbidopa as an anticancer agent for PCa.

Carbidopa, a peripheral decarboxylase inhibitor, has been reported to have an anticancer effect in a variety of tumors, such as breast cancer, pancreatic cancer, and lung cancer^7,19^. It was first reported in melanoma in 1983^20^. Recent studies have provided strong evidence that AR determines the progression of PCa after hormone ablation therapy, since the AR plays an important role in the survival of this devastating disease^21^. Targeting AR signaling pathway is the molecular basis for hormonal therapy, which either inhibits androgen production or blocks AR function^22^. It has been reported that L-Dopa decarboxylase (DDC) is an AR coactivator that increases in expression with disease progression, and is co-expressed with AR in prostate adenocarcinoma cells, where it may enhance AR activity. Carbidopa, as an inhibitor of DDC, can suppress DDC coactivation of AR and retard prostate tumor growth^23^. However, there may be additional mechanisms for Carbidopa in inhibiting PCa. In the present study, we have demonstrated that Carbidopa directly promotes the degradation of the AR protein, thus provides a new mechanism for the suppression of PCa by Carbidopa (Figs. 3a, b and 7g).

Aryl hydrocarbon receptor (AHR) is highly expressed in multiple organs and tissues, and there is increasing evidence that the AHR plays an important role in cellular homeostasis and disease^24,25^. AHR is expressed in multiple tumor types, cancer cell lines, and tumors from animal models. That said, the role of AHR signaling does not appear to be the same in all tumors. It exhibits a pro-oncogenic activity, enhancing growth, survival, and migration/invasion in colon cancer, gastric cancer, and androgen-independent PCas; in contrast, in breast cancer, liver cancer, pancreatic cancer, and androgen-dependent PCas, it induces tumor suppression characterized by growth inhibition, apoptosis, and decreased migration/invasion^26^. Thus, in PCa, the role of AHR depends on whether it is AR dependent or AR independent. In LNCaP and VCAP cells (AR dependent), activation of AHR inhibits cell proliferation through the degradation of AR. AHR agonists induces MMP-9 in androgen-insensitive PC3 and DU145 cells (AR independent), suggesting that the chemotherapeutic activity of AHR agonists may primarily be associated with androgen-sensitive PCa^27^. It was interesting that Ogura et al.^7^ used 1–10 µM of Carbidopa in pancreatic cancer cells to activate AHR; in our studies, higher concentrations were needed for the anticancer effcts in PCa cells. To determine the relevance of AHR for the observed anticancer effects of Carbidopa, we monitored AHR expression levels in BxPC3 and LNCaP cells. We found relatively less expression of AHR in LNCaP cells (Supplementary Fig. 6). It is possible that the level of expression of AHR could determine the concentration of Carbidopa needed to elicit its anticancer effect. Initial studies investigated AHR–AR cross talk in PCa cells, and showed that TCDD (an AHR ligand) inhibited basal and androgen-induced growth and cell cycle progression (G0/G1 to S-phase arrest)^28,29^. Here, we show the AHR–AR cross talk using Carbidopa, an already FDA-approved drug; our studies suggest that Carbidopa could show efficacy in the treatment of PCa.

The ubiquitin-proteasome system, which regulates cellular protein degradation, plays a pivotal role in cellular homeostasis^30^. Previous studies have shown that AHR acts as an E3 ubiquitin ligase to modulate steroid receptor functions^31^. AHR has recently been shown to promote the proteolysis of ER/AR through assembling a ubiquitin ligase complex, CUL4-B/AhR^32^. Our studies show that AR is a substrate for AHR-mediated ubiquitination and consequent degradation via proteasome. This is evident in LNCaP cells where Carbidopa activates AHR, and promotes AR ubiquitination and its degradation (Fig. 3). Co-immunoprecipitation shows clearly that AHR and AR interact with each other, a step necessary for AR to serve as a substrate for AHR-mediated ubiquination (Fig. 5c). These data also explain why AR mRNA levels are not altered in LNCaP cells in response to Carbidopa treatment, when AR protein levels are decreased.

Our data have proven that Carbidopa has potential as an anticancer agent for PCa. Our in vivo studies have shown that nude mice treated by i.p. injection of 7.5 mg/kg Carbidopa twice every day inhibited the growth of tumor. The recommended dose for Carbidopa in humans for the treatment of PD is 200 mg/day, but the drug is safe even at a dose as high as 450 mg/day^33^. In this study, the effective dose in mice translated to a human dose is ~100 mg/day (allometric scaling calculations from the FDA Draft guidelines)^34,35^. Thus, the concentration of Carbidopa that is effective as anticancer agent in the present study is below the FDA-recommended dose for humans, and at this dose, the drug is safe with no known undesirable side effects. Since Carbidopa is already FDA-approved, it could be fast-tracked in clinical trials to evaluate its efficacy for the treatment of PCa.

In sum, our data demonstrated that the anticancer effect of Carbidopa on PCa could be mainly owing to its role in activating AHR and resultant ubiquitination and degradation of AR (Fig. 8). These studies uncover a novel, hitherto unknown, pharmacologic function of Carbidopa that is relevant to the use of this drug for the treatment of PCa.

## Materials and methods

### Cell culture

LNCaP, VCAP, PC3, and DU145 cells, purchased from the American Type Culture Collection (Manassas, VA, USA), were cultured at 37 °C in a humidified atmosphere of 5% CO_2_–95% air. For routine maintenance, the cells were grown in RPMI-1640 medium supplemented with 10% fetal bovine serum (FBS), 50 mg/L streptomycin and 5000 units/L penicillin, and were passaged with trypsinization every fourth day. For assays, cells were plated at a density of 5000 cells/cm^2^ in multiwell culture plates, and cultured in phenol red-free RPMI-1640 medium. After in culture for 24 h, the cells were washed once with phosphate-buffered saline (PBS) and then treated with Carbidopa (Sigma, St. Louis, MO, USA) for 24 h.

### Cell proliferation assay

Cell proliferation was measured by MTT assay. Briefly, cells were seeded in a 96-well microplate at 2 × 10^3^ cells/well. After 12 h, the cells were incubated for 24 h in the medium supplemented with 0–200 μM Carbidopa. Then, the medium was replaced with fresh culture medium containing MTT (5 mg/mL in PBS; Solarbio, Beijing, China) and incubated for 4 h. The reaction product (formazan crystals) was dissolved in 150 μL of dimethyl sulfoxide (Sigma, St. Louis, MO, USA). Cell viability was assessed by measuring absorbance at 490 nm using a multiwell plate reader.

### Colony formation assay

Cells were seeded in six-well plate. After 12 h, the cells were incubated in the medium supplemented with Carbidopa. The medium was changed every 2 or 3 days. When the culture reaches 60–70% confluence, the cells were fixed with ice-cold 100% methanol for 20 min. Then, the cells were incubated with 0.1% crystal violet (Solarbio, Beijing, China) for 2 h. Subsequently, the cells were washed three times with tap water and then were imaged.

### Cell migration assay

The cells were seeded into six-well plates and incubated in the medium with 2.5% FBS until grown to full confluency, then scraped by a sterile 200 μL pipette tip. The medium was replaced with PBS, and the wound gap was photographed with an inverted microscope with a digital camera (Leica DM 14000B, Wetzlar, Germany) at 0 h and 24 h.

### Apoptosis analysis

Cell apoptosis was determined by flow cytometry and TUNEL staining. For flow cytometry assay, the cells were stained with Annexin V/FITC–propidium iodide using Apoptosis Detection kit (BD Biosciences, San Jose, CA, USA) and analyzed by flow cytometry. For TUNEL assay, the cells and the tumor tissues were stained with an In Situ Cell Death Detection Kit (Roche, Basel, Switzerland) according to the manufacturer’s protocol. The stained cells were imaged with a confocal laser-scanning microscope (TCS SP8, Leica, Wetzlar, Germany). One hundred cells per field were counted, and the percentage of TUNEL-positive cells was calculated.

### RNA isolation and PCR

Total RNA was extracted from LNCaP cells by using TRIZOL Reagent (Invitrogen, Carlsbad, CA, USA) according to the manufacturer’s instructions. Next, total RNA (2 µg) was reverse-transcribed into cDNA by using GoScript Reverse Transcription Kit (Promega, Madison, WI, USA). Subsequently, RT-PCR was conducted in Thermal Cycler (T100, Bio-Rad, Hercules, CA, USA). PCR products were electrophoresed in agarose gel, visualized by EtBr-imaging (GelDoc XR+; Bio-Rad, Hercules, CA, USA). The quantitative PCR (qPCR) was conducted using FastSYBR Green qPCR Master Mix (Life Technologies) and Viia7 real-time PCR machine (Thermo Fisher Scientific). Changes in expression levels were calculated as the ratio of the treated sample to control samples. All samples were normalized to the expression of the GAPDH mRNA.

Primers used for PCR were as follows: *AHR:* 5′-TCAAATCCTTCCAAGCGGCA-3′ (Sense) and 5′-ACAGTTATCCTGGCCTCCGT-3′ (Antisense); *AR:* 5′-CAGGTGGAGGCAAATCTTCGT-3′ (Sense) and 5′-CCTGCAATCTGCCAATGG-3′ (Antisense); *ARNT:* 5′-GGTTTGGCAGCACACTCTATG-3′ (Sense) and 5′-ACAGTTATCCTGGCCTCCGT-3′ (Antisense); *CYP1A1:* 5′-CAAGGGGCGTTGTGTCTTTG-3′ (Sense) and 5′-GTCGATAGCACCATCAGGG-3′ (Antisense); *GAPDH:* 5′-CGGAGTCAACGGATTTGGTCGTAT-3′ (Sense) and 5′-AGCCTTCTCCATGGTGGTGAAGA-3′ (Antisense).

### Western blot

Briefly, lysates from cells and tumor tissues were prepared, and protein levels determined using the BCA assay (Bio-Rad, Hercules, CA). A total of 30 μg protein from each sample was resolved by SDS–PAGE on Tris-glycine gels and transferred to PVDF membrane. Membranes were blocked with 5% bovine serum albumin (BSA) in Tris-buffered saline containing 0.1% Tween 20 (TBST) and incubated with primary antibodies overnight at 4 °C. Membranes were washed three times for 5 min each with TBST, incubated in either HRP-goat-anti-mouse (ab6789, Abcam) or HRP-goat-anti-rabbit (ab6721, Abcam) secondary antibodies for 2 h at room temperature. Immunoreactive bands were visualized using Pierce ECL plus Western blotting substrate (32132, Thermo Fisher Scientifc). The primary antibodies used in the present study were against: AR (5153 S, Cell Signaling Technology, Boston, MA, USA), PSA (5365 S, Cell Signaling Technology), β-actin (4970 S, Cell Signaling Technology), c-Caspase-3 (9661 S, Cell Signaling Technology), Bax (2774 S, Cell Signaling Technology), Bcl-2 (15071 S, Cell Signaling Technology), ubiquitin (3936 S, Cell Signaling Technology), and AHR (ab190797, Abcam). The protein bands were analyzed using ImageQuant 5.2 software. The expression of β-actin was used as a loading control.

### Immunofluorescence staining

Immunofluorescence staining was performed for quantification of nuclear localization of AHR in LNCaP cells. Briefly, cells in chamber slides were fixed with 4% paraformaldehyde for 30 min and permeabilized with 0.5% Triton-X 100 (Solabio, Beijing, China) for 10 min at 37 °C. After washing, the cells were blocked in 5% BSA for 1 h, and incubated with primary rabbit antibody against AHR at 4 °C overnight. Cells were then washed and incubated with Alexa Fluor 488-conjugated anti-mouse IgG secondary antibody for 1 h. Cells were again washed with PBS, and the cell nuclei were stained with DAPI for 15 min. Photo capture was performed by the confocal laser-scanning microscope (TCS SP8, Leica). The target visual field was randomly selected, and the nuclear localization of AHR and DAPI cells were observed under the microscope.

### Co-immunoprecipitation

LNCaP cells were lysed with IGEPAL CA-630 buffer (50 mM Tris-HCl, pH 7.4, (Sigma, T5030), 1% IGEPAL CA-630 (Sigma, I8896), 10 mM EDTA, 150 mM NaCl, 50 mM NaF, 1 μM leupeptin (Sigma, L5793), and 0.1 μM aprotinin (Sigma, SRE0050)). Primary antibody was covalently immobilized on protein A/G agarose using the Pierce Crosslink Immunoprecipitation Kit according to the manufacturer’s instructions (Thermo Scientific, 26147). Samples were incubated with immobilized antibody beads for at least 2 h at 4 °C. Cell lysates were also subjected to immunoprecipitation with either mouse IgG isotype control (Cell Signaling Technology, 5415) or rabbit IgG isotype control (Cell Signaling Technology, 3900), depending on the immunoglobulin type of primary antibody. After immunoprecipitation, the samples were washed with TBS five times. They were then eluted with glycine-HCl (0.1 M, pH 3.5) and the immunoprecipitates were subjected to immunoblotting using specific primary antibodies.

### PCa xenografts

Seven-week-old BALB/c nude male mice (18–22 g; *N* = 12) were purchased from Vital River Laboratories (Beijing, China). Animals were housed at a constant room temperature with a 12/12-hr light/dark cycle and a standard rodent diet was fed to them. The 12 mice were randomly divided into two experimental groups, and six in each group. The group allocation during the experiment was blind to the investigators. LNCaP cells were subcutaneously injected into the right flank of each mouse with 1 × 10^7^ cells in 0.1 mL PBS. Once tumors reached to a volume of 100 mm^3^, mice were treated by i.p. injection of 7.5 mg/kg Carbidopa or the same amount of vehicle twice every day. All mice were treated for 14 days. The tumor length (l) and width (w) were measured to calculate the tumor volumes (V = 0.5 × l × w^2^) at the indicated time points. At the end of study, mice were sacrificed after being anesthetized by i.p. injection with pentobarbital (50 mg/kg). Tumor specimens were harvested and weighed. Samples were then processed for histology and protein assays. All animal studies were conducted according to the Guidelines for Animal Experimentation of Wenzhou Medical University, and the protocol was approved by the Animal Ethics Committee of Wenzhou Medical University.

### Immunohistochemistry

Harvested tumor tissues were fixed in 10% formalin and embedded in paraffin. Specimens were sectioned at 5 µm thickness. Tumor sections were stained using routine immunohistochemical techniques with primarily antibodies against Ki67 (ab15580, Abcam), and HRP-conjugated secondary antibodies were used for detection. Finally, tumor specimens were also stained with hematoxylin and then were observed under the microscope.

### Statistical analysis

Data are expressed as means ± SEM. Statistical differences were assessed with the unpaired two-tailed Student’s *t*-test for two experimental groups and one-way ANOVA for multiple groups with SPSS software. Bonferroni’s post hoc testing was employed after ANOVA for testing for significant differences between groups. A two-tailed *p*-value of <0.05 was considered statistically significant. Statistical analyses were done using GraphPad Prism (GraphPad Software).

## Supplementary information

Supplemental materials
