# Supplementary material for: Carbidopa suppresses prostate cancer via aryl hydrocarbon receptor-mediated ubiquitination and degradation of androgen receptor
Source: Oncogenesis. 2020 May 13;9(5):49. doi: 10.1038/s41389-020-0236-x (PMC7220950; doi:10.1038/s41389-020-0236-x)
Supplement: Supplementary file 1 — Supplemental materials [file 41389_2020_236_MOESM1_ESM.docx]

**Supplement Information**


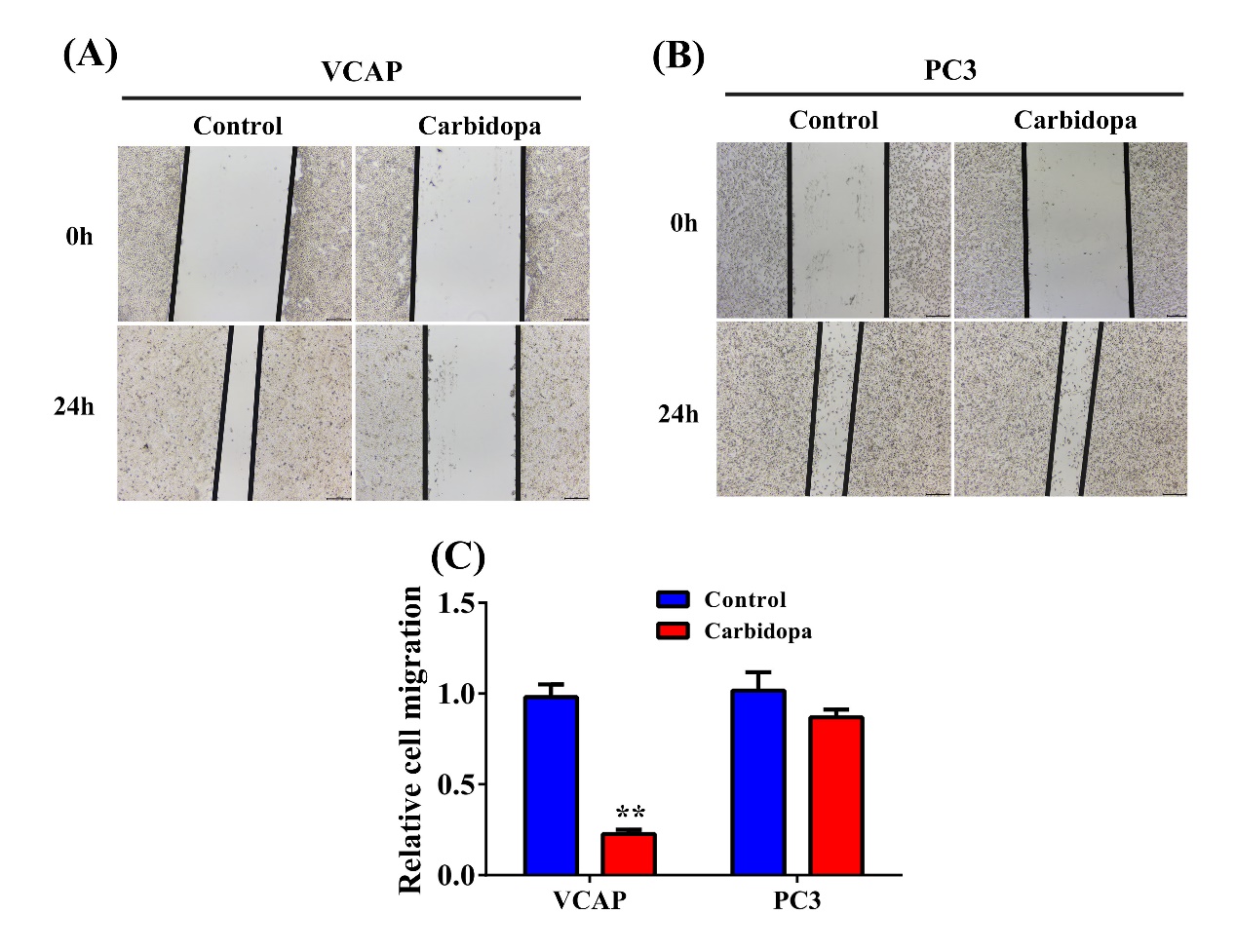


**Fig. S1. Carbidopa inhibits VCAP cells migration but has no effect on PC3 cells.** Migration assay analysis (A) VCAP and (B) PC3 cells with or without Carbidopa. (C) Quantitative analysis of cell migration in at least 3 separate fields; data are given as means ± SEM. **, p < 0.01 versus control.


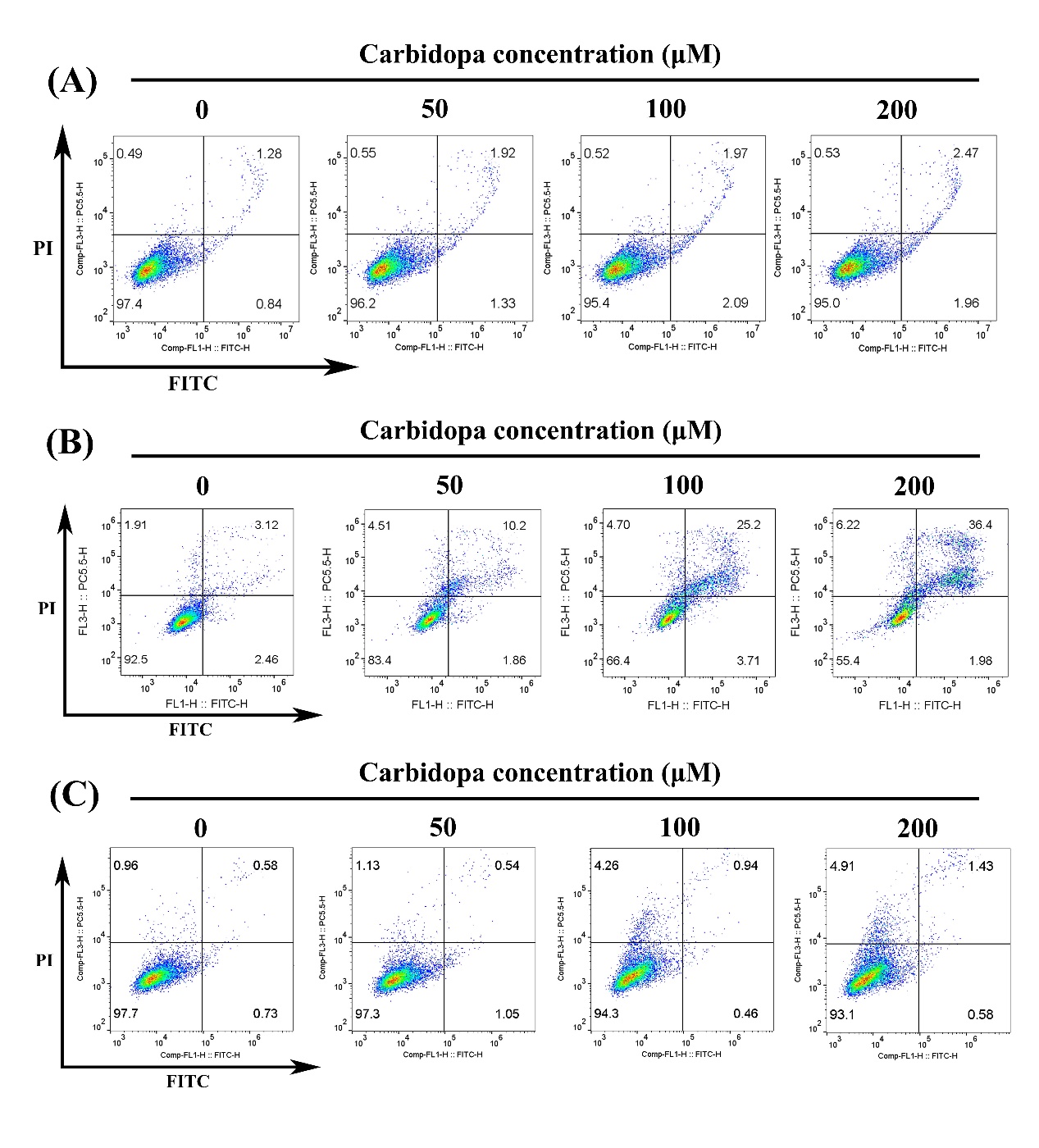


**Fig. S2. Carbidopa induced cell apoptosis in VCAP cells but not in PC3 and DU145 cells.** Flow cytometry analysis of apoptosis on (A) DU145, (B) VCAP and (C) PC3 cells with increasing doses of Carbidopa.


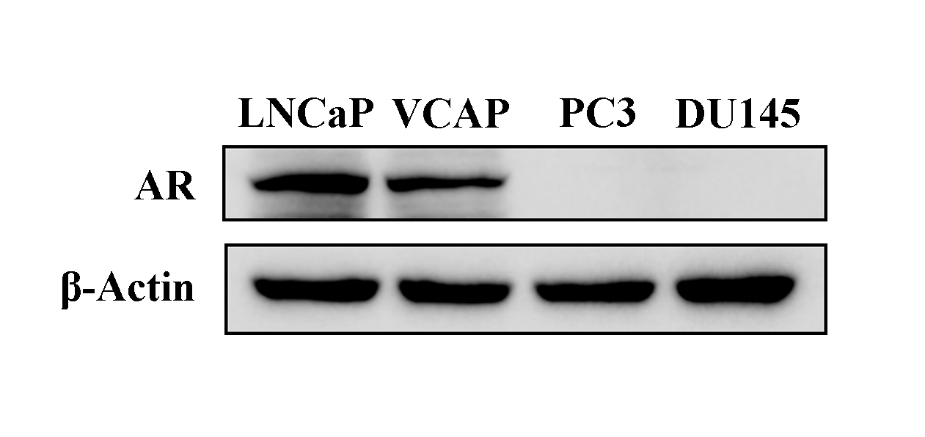


**Fig. S3.** **AR protein level in four prostate cell lines**


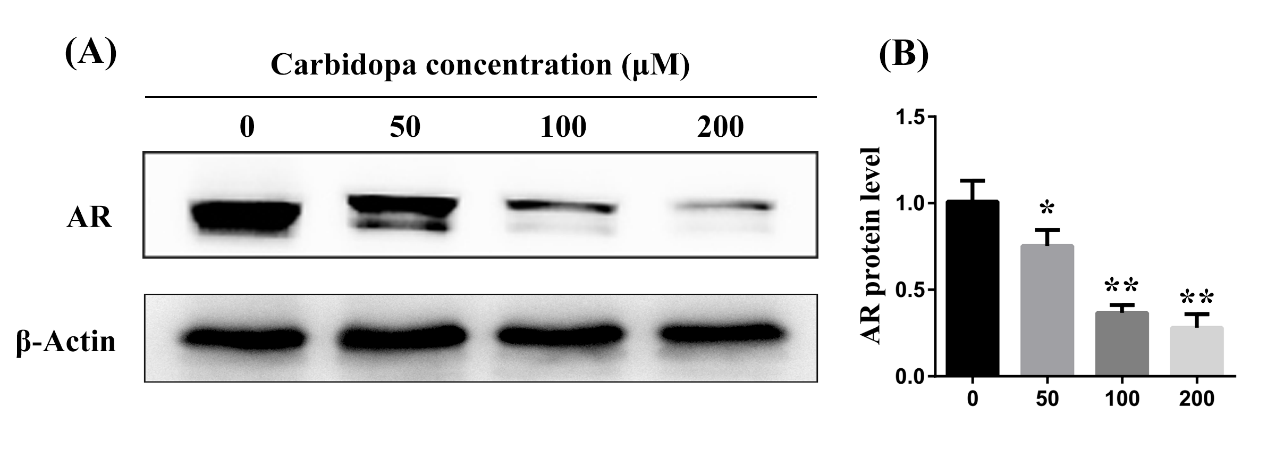


**Fig. S4. Carbidopa decreases AR protein level dose-dependently in VCAP cells.** (A) Immunoblot analysis of AR protein level. VCAP cells were treated increasing doses of Carbidopa for 24 h. (B) Quantitative analysis of each immunoblot; results were normalized to control and data are given as means ± SEM of 3 independent experiments. *, p < 0.05 versus control. **, p<0.01 versus control (no Carbidopa).


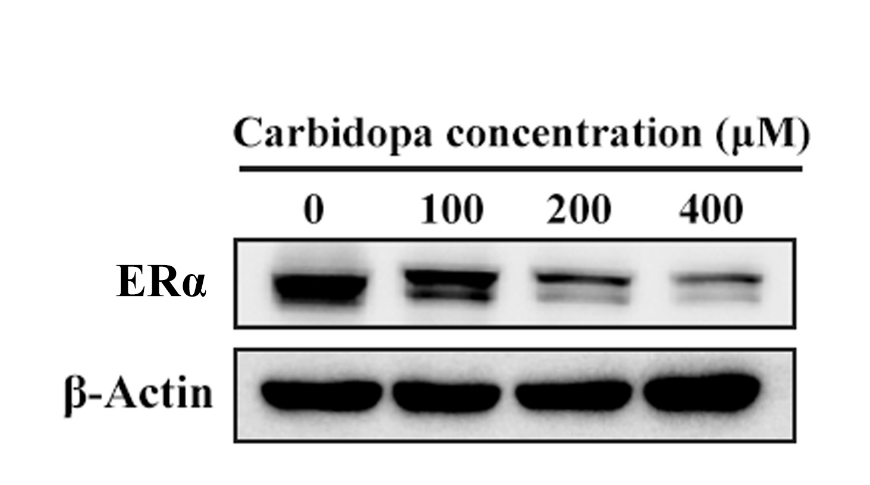


**Fig. S5. Effect of Carbidopa on ERα in MCF7 cells**


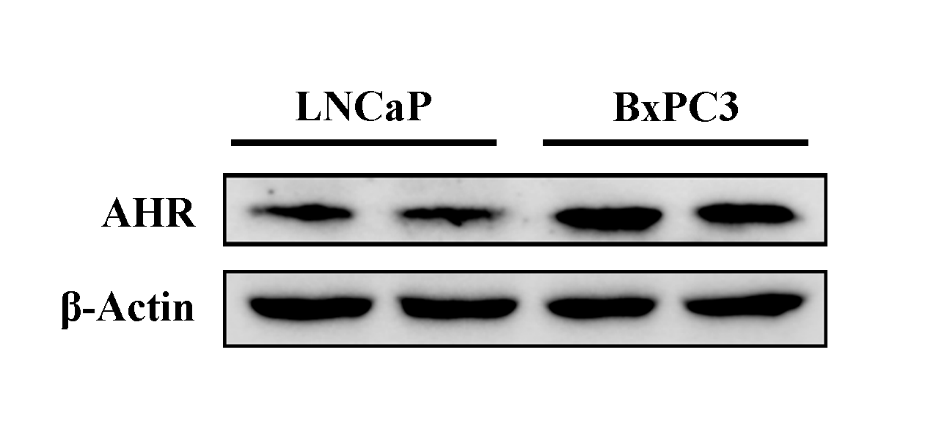


**Fig. S6.** **AHR protein level in LNCaP cells and BxPC3 cells**
